# Fireside Associations

**Published:** 1866-11

**Authors:** Ada Davidson


### FIRESIDE ASSOCIATIONS.

BY ADA DAVIDSON.

Give us reading—with a name which is a pleasant reminder of the social circle, and all the dear delights of home; recalling purest memories of all the most precious relations in private life, the care and protection, the advice and counsel we have received from age, the love and sympathy from equals, and the interest which invests all its associations.

He who has no cherished recollections clustering round a remembered fireside, has lived an unloved life, and is destitute of one of the strongest safeguards of virtue. A nation of happy firesides possesses within itself elements of strength, which can defy the attacks of iron-ribbed navies, or leagued battalions. It is a synonyme for the "only bliss which has survived the fall."

We welcome to our homes another monthly. The supply of such periodicals creates a demand. Every new painting, taken from the easel of genius, fresh and glowing with the warm beauties of Summer, or the wierd transformations of Winter, awakes the taste for its appreciations, and kindles a desire for new and varied productions of the pencil. Every grand oratorio fills some longing in the tuned ear, unsatisfied before, and incites to new efforts in the wonderful combination of harmonious sounds.

The shelves of costly libraries groan with ponderous philosophics and learned homiletics: we need something less cumbrous and expensive for daily use and isolated hours. We need these golden sands to fill up the interstices of our daily life, making it beautiful with these sterling truths; and we appreciate the efforts of those who sift them from their granite casings, and send them pure and glittering to our firesides.

Such literature is becoming a necessity of our intellectual nature; it should be as plentiful as air, as refreshing as the common air—the generous air environing us on every side, below, around, beneath us—not elevated above us in cloud-land, but close at hand, within our dwellings, for each moment's uses, for us to breathe, to live upon, which none, however wealthy, can monopolize, because it *is* common. It should be as pure, as life-giving, as the common light, the sun-light, not only dancing in upper ether, from star to star, nor painting in glory the sunset, or the aristocratic mountain-top, but coming at once with its golden radiance to the level of our daily walks and common needs, greening the sod beneath our feet, pouring its flood of blessing through the lowly casement as well as the painted oriel.

If we must eat, let our food be healthful, and not poisonous. If our thirst needs to be allayed, let us have water pure, fresh, and sparkling, from the crystal fount—not turbid, and muddy, and intoxicating. Our mental pabulum should be refined and strengthening. It is not necessary for knowledge to be valuable, that it should be written in foreign tongues, or in black letters, or enclosed in heavy lids within folio and quarto. The guinea, subdivided, is not the less pure gold, and answers more purposes in its more frequent circulation, and performs a thousand more offices of kindness than in its condensed form. Only let it be gold, sterling gold, and it cannot be too plentiful, nor gladden too many firesides.

